# Supplementary material for: Representation of women at American Psychiatric Association annual meetings over 10 years (between 2009 and 2019)
Source: PLoS One. 2022 Jan 25;17(1):e0261058. doi: 10.1371/journal.pone.0261058 (PMC8789168; doi:10.1371/journal.pone.0261058)
Supplement: S1 Table — (DOCX) [file pone.0261058.s001.docx]

**S1 Table. Comparison of the proportions of male and female speakers in different session types at the APA 2009 and 2019 annual meetings**

| **Sessions** | **2009** | | | | | **2019** | | | | | **Rate of change (%)** |
| --- | --- | --- | --- | --- | --- | --- | --- | --- | --- | --- | --- |
|  | **Men** | | **Women** | | **Total** | **Men** | | **Women** | | **Total** |  |
|  | **n** | **%** | **n** | **%** |  | **n** | **%** | **n** | **%** |  |  |
| **Presidential session** | 31 | 64.6 | 17 | 35.4 | 48 | 102 | 59.0 | 71 | 41.0 | 173 | 15.9 |
| **Master courses** | 9 | 64.3 | 5 | 35.7 | 14 | 17 | 81.0 | 4 | 19.0 | 21 | - 46.7 |
| **Special session** | 8 | 57.1 | 6 | 42.9 | 14 | 14 | 58.3 | 10 | 41.7 | 24 | - 2.8 |
| **Workshop** | 236 | 54.5 | 197 | 45.5 | 433 | 37 | 44.6 | 46 | 55.4 | 83 | 21.8 |
| **Symposiums** | 467 | 66.9 | 231 | 33.1 | 698 | 1090 | 53.8 | 936 | 46.2 | 2026 | 39.6 |
| **Courses** | 172 | 72.6 | 65 | 27.4 | 237 | 79 | 60.3 | 52 | 39.7 | 131 | 44.7 |
| **Other or not defined** | 3 | 75.0 | 1 | 25.0 | 4 | 16 | 44.4 | 20 | 55.6 | 36 | 122.4 |
