# Supplementary material for: Representation of women at American Psychiatric Association annual meetings over 10 years (between 2009 and 2019)
Source: PLoS One. 2022 Jan 25;17(1):e0261058. doi: 10.1371/journal.pone.0261058 (PMC8789168; doi:10.1371/journal.pone.0261058)
Supplement: S2 Table — (DOCX) [file pone.0261058.s002.docx]

**S2 Table. Comparison of the proportion of men and women by role at the APA 2009 and 2019 APA annual meetings**

| **Roles** | **2009** | | | | | **2019** | | | | | **Rate of change (%)** |
| --- | --- | --- | --- | --- | --- | --- | --- | --- | --- | --- | --- |
|  | **Men** | | **Women** | | **Total** | **Men** | | **Women** | | **Total** |  |
|  | **n** | **%** | **n** | **%** |  | **n** | **%** | **n** | **%** |  |  |
| **Chair** | 241 | 60.4 | 158 | 39.6 | 399 | 372 | 53.6 | 322 | 46.4 | 694 | 17.2 |
| **Director** | 92 | 79.3 | 24 | 20.7 | 116 | 35 | 74.5 | 12 | 25.5 | 47 | 23.4 |
| **Faculty** | 88 | 65.2 | 47 | 34.8 | 135 | 64 | 57.1 | 48 | 42.9 | 112 | 23.1 |
| **Discussant** | 16 | 72.7 | 6 | 27.3 | 22 | 52 | 59.1 | 36 | 40.9 | 88 | 50.0 |
| **Presenter** | 489 | 63.1 | 286 | 36.9 | 775 | 831 | 53.5 | 721 | 46.5 | 1552 | 25.9 |
| **Other or not defined** | 0 | 0 | 1 | 100.0 | 1 | 1 | 100.0 | 0 | 0 | 1 | - |
