# Supplementary material for: Representation of women at American Psychiatric Association annual meetings over 10 years (between 2009 and 2019)
Source: PLoS One. 2022 Jan 25;17(1):e0261058. doi: 10.1371/journal.pone.0261058 (PMC8789168; doi:10.1371/journal.pone.0261058)
Supplement: S3 Table — (DOCX) [file pone.0261058.s003.docx]

**S3 Table. Comparison of the proportion of men and women among speakers by topic at the 2009 and 2019 APA annual meetings**

| **Topics^a^** | **2009** | | | | | **2019** | | | | | **Rate of change (%)** |
| --- | --- | --- | --- | --- | --- | --- | --- | --- | --- | --- | --- |
|  | **Men** | | **Women** | | **Total** | **Men** | | **Women** | | **Total** |  |
|  | **n** | **%** | **n** | **%** |  | **n** | **%** | **n** | **%** |  |  |
| **Addiction Psychiatry** | 80 | 60.2 | 53 | 39.8 | 133 | 97 | 69.3 | 43 | 30.7 | 140 | -22.9 |
| **Child and Adolescent Psychiatry** | 36 | 48.6 | 38 | 51.4 | 74 | 76 | 49.0 | 79 | 51.0 | 155 | -0.7 |
| **Consultation liaison Psychiatry** | 82 | 65.1 | 44 | 34.9 | 126 | 57 | 55.9 | 45 | 44.1 | 102 | 26.3 |
| **Diversity and Health Equity** | 130 | 55.6 | 104 | 44.4 | 234 | 102 | 44.9 | 125 | 55.1 | 227 | 23.9 |
| **Resident, Fellows and Medical Student** | 58 | 47.9 | 63 | 52.1 | 121 | 125 | 43.3 | 164 | 56.7 | 289 | 9.0 |
| **Forensic Psychiatry** | 83 | 70.9 | 34 | 29.1 | 117 | 104 | 57.5 | 77 | 42.5 | 181 | 46.4 |
| **Geriatric Psychiatry** | 23 | 60.5 | 15 | 39.5 | 38 | 26 | 48.1 | 28 | 51.9 | 54 | 31.4 |
| **Other Topics** | 452 | 70.8 | 186 | 29.2 | 638 | 816 | 55.9 | 645 | 44.1 | 1461 | 51.4 |

^a^ Two topics were assigned for 148 (3.8%) presentations.
