# Supplementary material for: Representation of women at American Psychiatric Association annual meetings over 10 years (between 2009 and 2019)
Source: PLoS One. 2022 Jan 25;17(1):e0261058. doi: 10.1371/journal.pone.0261058 (PMC8789168; doi:10.1371/journal.pone.0261058)
Supplement: Attachment — Submitted filename: Reponse to Reviewers.docx [file pone.0261058.s005.docx]

RESPONSE TO REVIEWERS

We do need to highlight whether there is an existent sex disparity or not. Hence, this I think that topic is relevant in terms of academia.

- Response: Thank you for the useful comments to enhance the quality of the paper.

However, the topic does not represent the paper well. I would not use the word 'equity' here.

- Response: We understand that sex disparity is a relevant topic. As suggested by the reviewer, we changed the word ‘equity’ in the text, and we replaced it in most cases by the word ‘disparity’.

We also changed the title of the manuscript as follows: “Representation of women at American Psychiatric Association annual meetings over 10 years (between 2009 and 2019).” Indeed, this title is consistent with the primary objective of our work that was to evaluate the evolution of female speakers representation at APA annual meetings over ten years. In our paper, we highlighted an overall increase of female speakers in 2019 compared to 2009. We also showed that women were still underrepresented in leadership roles and honorific sessions in 2019.

We also changed the wording where appropriate in the manuscript, replacing the word ‘equity’ in most cases by the word ‘disparity’.

The introduction could have more updated references relating to sex differences in academia. There are several recent papers published on this topic.

- Response: As suggested, we updated our references. We added more recent references in the Introduction and Discussion sections as suggested.

We added the following references:

15. Arora A, Kaur Y, Dossa F, Nisenbaum R, Little D, Baxter NN. Proportion of Female Speakers at Academic Medical Conferences Across Multiple Specialties and Regions. JAMA 2020;3(9):e2018127.

16. Cohen-Rosenblum AR, Bernstein JA, Cipriano CA. Gender Representation in Speaking Roles at the American Association of Hip and Knee Surgeons Annual Meeting: 2012-2019. The Journal of Arthroplasty. 2021;S0883540321000383.

17. Harris KT, Clifton MM, Matlaga BR, Koo K. Gender Representation Among Plenary Panel Speakers at the American Urological Association Annual Meeting. Urology. 2021;150:54‑8.

32. Schroeder E, Rochford C, Voss M, Gabrysch S. Beyond representation: women at global health conferences. The Lancet. 2019;393:1200‑1.

The methodology is weak. The authors should use hierarchical regression technique in their statistical analysis. That would give more insight into the results once partial pooling is used. At present, frequencies and chi square analysis have been applied in the analysis. This analysis does not do justice to the objective of the paper. The discussion could be made stronger by using updated references.

- Response: We followed your recommendations. As requested, we performed new analyses.

We added the following to the Statistics section: “We performed a multivariable analysis by logistic regression, evaluating the probability of female participation according to role, type of session, topic and APA year (2019 vs 2009). The descriptive analysis was conducted with SPSS (IBM, Inc., Armonk, NY). The multivariable analysis was conducted using R version 4.0.2 (www.R-project.org). All the tests used were two-tailed. Statistical significance was defined as p-value<0.05.”

We added the following to the Results section: “Results of the multivariable analysis are presented in Table 2. The probability of female participation increased between 2009 and 2019, with an Odds Ratio of 1.580 (95%CI 1.351-1.850, p<.001). The Area Under the Curve (AUC) was 0.603. No calibration problems were found, the Hosmer-Lemeshow Goodness-of-fit test was not significant (p=0.19).”

We added the Table 2: Multivariable analysis by logistic regression modelling the probability of female participation according to APA session.

1. Please ensure that your manuscript meets PLOS ONE's style requirements, including those for file naming. The PLOS ONE style templates can be found at https://journals.plos.org/plosone/s/file?id=wjVg/PLOSOne_formatting_sample_main_body.pdf and https://journals.plos.org/plosone/s/file?id=ba62/PLOSOne_formatting_sample_title_authors_affiliations.pdf

- Response: We made the appropriate changes according to the guidelines.

2. Please improve statistical reporting and refer to p-values as "p<.001" instead of "p<.0001". Our statistical reporting guidelines are available at <https://journals.plos.org/plosone/s/submission-guidelines#loc-statistical-reporting>

- Response: We made the appropriate changes in statistical reporting as required.

3. Please include your tables as part of your main manuscript and remove the individual files. Please note that supplementary tables (should remain/ be uploaded) as separate "supporting information" files

- Response: As requested, we included the Tables in the main manuscript. We also added “supporting information” files as required.

4. Thank you for stating the following in the Competing Interests section: CH reports personal fees from EISAI, Janssen, Lundbeck, Otsuka and UCB. WEH reports personal fees from EISAI, Janssen, Lundbeck, Otsuka, UCB, Roche and Chugai.

Please confirm that this does not alter your adherence to all PLOS ONE policies on sharing data and materials, by including the following statement: "This does not alter our adherence to PLOS ONE policies on sharing data and materials.” (as detailed online in our guide for authors http://journals.plos.org/plosone/s/competing-interests). If there are restrictions on sharing of data and/or materials, please state these. Please note that we cannot proceed with consideration of your article until this information has been declared.

Please include your updated Competing Interests statement in your cover letter; we will change the online submission form on your behalf.

Please know it is PLOS ONE policy for corresponding authors to declare, on behalf of all authors, all potential competing interests for the purposes of transparency. PLOS defines a competing interest as anything that interferes with, or could reasonably be perceived as interfering with, the full and objective presentation, peer review, editorial decision-making, or publication of research or non-research articles submitted to one of the journals. Competing interests can be financial or non-financial, professional, or personal. Competing interests can arise in relationship to an organization or another person. Please follow this link to our website for more details on competing interests: <http://journals.plos.org/plosone/s/competing-interests>

- Response: As requested, we added a Competing Interests section reporting the following: “CH reports personal fees from EISAI, Janssen, Lundbeck, Otsuka and UCB. WEH reports personal fees from Air Liquide, EISAI, Janssen, Lundbeck, Otsuka, UCB, Roche and Chugai. This does not alter our adherence to PLOS ONE policies on sharing data and materials.”

We confirm that the reported competing interests do not alter our adherence to PLOS ONE policies on sharing data and materials. We also removed the competing interests section from the our manuscript. We included our updated Competing Interests statement in your cover letter.

5. Please amend your list of authors on the manuscript to ensure that each author is linked to an affiliation. Authors’ affiliations should reflect the institution where the work was done (if authors moved subsequently, you can also list the new affiliation stating “current affiliation:….” as necessary).

- Response: We made the appropriate changes as required.

6. Please ensure that you refer to Figure 1-2 in your text as, if accepted, production will need this reference to link the reader to the Figure.

- Response: We added the references for the Figures in the text. We also changed the way we presented the Figures in line with PLOS ONE's style requirements.

7. Please include captions for your Supporting Information files at the end of your manuscript, and update any in-text citations to match accordingly. Please see our Supporting Information guidelines for more information: <http://journals.plos.org/plosone/s/supporting-information>.

- Response: We added captions for supporting information files at the end of the manuscript. We also added a separate file.

**Review Comments to the Author**

Please use the space provided to explain your answers to the questions above. You may also include additional comments for the author, including concerns about dual publication, research ethics, or publication ethics. (Please upload your review as an attachment if it exceeds 20,000 characters).

**Reviewer #1:**

This study addressed an interesting and nice topic, and especially for the first time in psychiatry. It was appropriately planned and analyzed as well as was written in a well-fashioned manner. The aim of the study, methodology, and conclusions were perfectly synchronized.

Minor suggestion: The authors mentioned, “National demographic data were obtained from the website of the Association of American Medical Colleges for 2007 and 2017 as data for 2009 and 2019 were not available”. It represents that, these were the latest data, that is data for 2008 and 2018 were also not available. I would like to suggest adding this to the statement.

- Response: Many thanks for the proposition. As proposed, we added the following sentence in the manuscript: “Data for 2008 and 2018 were also not available.”

**Reviewer #2: Comments**

1. Is the title is a current public health issue? NO

- Response: Our work addressed the evolution of the representation of women in the most attended annual psychiatry meeting. It also questioned women actual place in academic psychiatry in general. Thus, this work is indeed not at public health issue but , we believe, an important society issue. Hence, we think that topic is relevant in terms of academia.

2. Is the objective is clear and measurable? NO

- The goal of this work was to explore the evolution of women representation among speakers of the APA annual meetings over 10 years. To measure the evolution of women speakers, we analyzed the evolution of the proportion of women speakers at the APA annual meeting in 2009 and 2019. In order to explore this evolution, we compared the sex distribution of the speakers at the APA meeting in 2019 to the sex distribution of the speakers at the APA meeting in 2019. A variation of the sex distribution was highlighted with a p value< 0.05.

3. Is the method clear and explained well? NO

- Response: We used the programs of the 2009 and 2019 APA annual meetings to extract the data. Variables collected for each speaker were: sex, role during their intervention, type of the session they took part in and topic of the session.

3.1. Who is your study population?

- Response: Our study population consisted of the speakers at the 2009 and 2019 APA annual meetings.

3.2. What is your study design?

- Response: Our study was designed using a retrospective, cross-sectional methodology. We added that in our manuscript.

3.3. What is sampling technique?

- Response: No sampling was performed. We collected the data for all the speakers of the 2009 and 2019 APA annual meetings.

3.4. What is sample size?

- Response: Our study population was of 1093 and 1762 speakers at the 2009 and 2019 APA meetings respectively. We mentioned it in the results.

3.5. What about exclusion and inclusion criteria?

- Response: Speakers at the APA 2009 or/and 2019 meeting were included in this work. Speakers who participated only in poster presentations were excluded. Sessions which did not exist in 2009 and 2019 were not taken into consideration as well as the speakers who took part in it.

3.6. What is dependent variable?

- Response: Not relevant here.

4. Is your data is primarily or secondary? If primarily, how you collect through ten years? Or if secondary data, explain it also.

- Response: We did not collect data through ten years. We collected the data of 2009 and 2019 APA annual meeting programs.

In the programs, the sex of the speakers was not mentioned. We identified the sex of the speakers using the speaker’s name mentioned in the program combined with an internet search. From the meetings programs, we then extracted the type of sessions in which the speaker presented, the speaker’s role in the session and the topic of the session. All these data were compiled in an excel file. To avoid any mistake, the data was reviewed by two of the authors.

5. The recommendation needs major revision. Based on specific, measurable, time bound and reasonable.

- Response: Thank you for the useful comments to enhance the quality of the paper.
