# Supplementary material for: Representation of women at American Psychiatric Association annual meetings over 10 years (between 2009 and 2019)
Source: PLoS One. 2022 Jan 25;17(1):e0261058. doi: 10.1371/journal.pone.0261058 (PMC8789168; doi:10.1371/journal.pone.0261058)
Supplement: Attachment — Submitted filename: Referee Report PLOS ONE.pdf [file pone.0261058.s006.pdf]

Referee Report

PLOS ONE, PONE-D-20-40415R1

Representation of women at American Psychiatric Association annual meetings over 10 years (between 2009 and 2019)

A. Summary

This paper studies the change in women's representation in academic Psychiatry, which is an important and timely topic, by comparing the female share of women in the American Psychiatric Association annual meetings in 2009 versus 2019.

B. Overall takeaway

This paper finds that the percentage of female speakers was larger in 2019 than 2009, which implies that the representation of women at the APA annual meetings has increased during the period.

C. Major comments

1. Statistical method and interpretation

As the authors responded to reviewer #2, the *population* of this study is "the speakers at the 2009 and 2019 APA annual meetings," and the data of this study are not *sample* data, but *population* data: "We collected the data for *all* the speakers of the 2009 and 2019 APA annual meetings." Here, the parameters of interest are the percentages of women of all the speakers in the meetings, which can be found by simply calculating the percentage using the population data the authors have. In other words, the parameters of interest are *known*, so it is inappropriate to perform any statistical estimation (i.e. z-test, Chi2 analysis, p-value, etc.), which is the process to use sample estimates to approximate the value of *unknown* population parameters. See similar previous papers (e.g. Gerull et al. 2020, Sleeman et al. 2019, and May and Dimand 2019) which simply report the number/percentage of women without any tests and perform statistical tests only when they need to estimate some unknown parameters.

In the result section, the authors repetitively report estimates, p-values, and statistical significance of the estimates. For the reasons I mentioned above, however, I am neither persuaded that these statistical analyses in this study are well performed, nor that the interpretation and discussion of the results are presented in an appropriate/intelligible fashion.

2. Logistic regression

The authors mechanically report the result of the logistic regression without any interpretation. I cannot find any consideration as to why the explanatory variables are chosen or what the estimates mean. The authors added this analysis responding to a previous reviewer who suggested to use a hierarchical regression technique, but the logistic regression was not performed as such.

3. Suggestion

This paper would be more substantial if the authors went beyond the current research question: whether the percentage of women in the APA meetings increased between 2009 and 2019. The question would be easily answered by simply calculating the percentage of women in the data without any statistical inferences. A possible question which can be further studied with the data is which factor explains the increase in the women's representation (this is the question where statistical estimation is really needed). Although the authors suggest some possible explanations in the discussion section, they are weakly supported by the data. For example, an interesting question left for future research is "the impact of the sex of the chair on the choice of speaker at the APA annual meetings," as suggested by the authors in "Strengths and limitations." If the data provide the information on chairs of each APA meeting session, the authors would be able to regress the percentage of women in a session on the gender of the chair of the session controlling for other explanatory variables.

D. <u>Minor comments</u>

1. Consistency with the national demographic data

The authors could provide more empirical analyses, especially in terms of the limitations of this paper mentioned in the subsection "Strengths and limitations." For example, one of the limits is that the speakers of the APA meetings are not necessarily U.S. researchers; they also consist of researchers from other countries. The authors could reduce this concern by collecting data on the speakers' institutions (as the authors collected the gender of the speakers) to determine whether the speakers working within or without the U.S.

2. Referring to figures

Although a previous reviewer suggested to "ensure that you refer to Figure 1-2 in your text," such figures have not been explicitly referred to by the text. The authors could mention figures 1-3 somewhere in the subsections "Roles," "Topics," or "Sessions" of the Results section.

E. Citations

Katherine M. Gerull, Brandon Malik Wahba, Laurel M. Goldin, Jared McAllister, Andrew Wright, Amalia Cochran, Arghavan Salles. Representation of women in speaking roles at surgical conferences. *The American Journal of Surgery*. 2020;220(1):20-26.

Katherine E. Sleeman, Jonathan Koffman, Irene J. Higginson. Leaky pipeline, gender bias, self-selection or all three? A quantitative analysis of gender balance at an international palliative care research conference. *BMJ Supportive & Palliative Care.* 2019;9:146-148.

Ann Mari May, Robert W. Dimand. Women in the Early Years of the American Economic Association: A Membership beyond the Professoriate Per Se. *History of Political Economy.* 2019;51(4):671–702.
